# Soluble CD23 Levels are Inversely Associated with Atopy and Parasite-Specific IgE Levels but Not with Polyclonal IgE Levels in People Exposed to Helminth Infection

**DOI:** 10.1159/000346545

**Published:** 2013-05-14

**Authors:** Nadine Rujeni, Norman Nausch, Nicholas Midzi, Reginald Gwisai, Takafira Mduluza, David W. Taylor, Francisca Mutapi

**Affiliations:** ^a^Institute of Immunology and Infection Research, Centre for Immunity, Infection and Evolution, School of Biological Sciences, Edinburgh, UK; ^b^Division of Pathway Medicine, School of Biomedical Studies and Edinburgh Infectious Diseases, University of Edinburgh, Ashworth Laboratories, Edinburgh, UK; ^c^National Institute of Health Research, Murehwa, Zimbabwe; ^d^Department of Biochemistry, University of Zimbabwe, Harare, Murehwa, Zimbabwe; ^e^Murehwa Hospital, Murehwa, Zimbabwe

**Keywords:** Atopy, Schistosomiasis, Helminth, CD23, IgE

## Abstract

**Background:**

Protective acquired immunity against helminths and allergic sensitisation are both characterised by high IgE antibody levels. Levels of IgE antibodies are naturally tightly regulated by several mechanisms including binding of the CD23 receptor. Following observations that helminth infections and allergic sensitisation may co-present, the current study aims to investigate the relationship between the soluble CD23 (sCD23) receptor, parasite-specific IgE responses and allergic sensitisation in people exposed to the helminth parasite *Schistosoma haematobium*.

**Methods:**

A cohort of 434 participants was recruited in two villages with different levels of *S. haematobium* infection in Zimbabwe. Serum levels of the 25-kDa fragment of sCD23 were related to levels of schistosome infection intensity, allergen (house dust mite, HDM) and schistosome-specific IgE, total IgE and skin sensitisation to HDM.

**Results:**

sCD23 levels rose significantly with schistosome infection intensity but declined significantly with schistosome-specific IgE levels. Furthermore, sCD23 levels were negatively associated with skin sensitisation and IgE reactivity against HDM, but showed no relationship with total IgE.

**Conclusion:**

The results are consistent with the suppression of parasite and allergen-specific IgE levels by sCD23. Further mechanistic studies will determine the relevance of this potential regulatory mechanism in the development of helminth-specific immune responses in atopic individuals.

## Introduction

Helminth infections, including schistosomiasis, and atopic disorders are characterised by high levels of IgE antibodies. Helminth-specific IgE responses are protective [1,2,3,4] while high allergen-specific IgE levels are associated with the clinical manifestations of allergy [5,6,7,8]. In both cases, these IgE responses have been associated with increased CD23 expression [9,10]. sCD23 has been suggested as a diagnostic marker in a range of diseases [11]. The CD23 (BLAST-2 or low affinity FcεRII) is a 45-kDa transmembrane low affinity IgE receptor found on the surface of naïve IgM+ IgD+ B cells [12]. It also appears on the cell surface of macrophages, platelets, monocytes and eosinophils [13]. It is characterised by three lectin domains (heads) bound onto the membrane by a triple α-helical ‘tail’. The binding of IgE on membrane-bound CD23 is thought to inhibit the Cε gene transcription and IgE class switch, thus regulating IgE synthesis [14,15]. However, the tail region of the CD23 can be cleaved by endogenous proteases, in the presence of IL-4 and IL-13, resulting in release of soluble CD23 (sCD23) fragments to the serum [14,16].

CD23 is capable of both enhancing and inhibiting IgE production depending on the site of its cleavage. Cleavage of the membrane CD23 may occur at several proteolytic sites by metalloproteinases [11] and the released fragments may undergo further cleavage [17], resulting in monomeric and trimeric forms of sCD23. The trimers enhance survival and differentiation of IgE-committed B cells [14] increasing IgE secretion by PBMCs, while the monomeric sCD23 (16 and 25 kDa) decrease IgE secretion by PBMCs [18,19,20].

An increase in circulating CD23+ B cells as well as soluble CD23 (sCD23) has been associated with resistance to schistosome re-infection [9], while CD23+ B cells were higher in asthmatic compared to non-asthmatic children [10]. In the mouse model, CD23 has been shown to have an inhibitory role in the development of allergic airway inflammation and airway hyperresponsiveness [21]. To date, however, there are no studies describing the relationship between sCD23 levels and allergen-specific IgE levels concurrently with parasite-specific IgE levels. Understanding this relationship is important for determining mechanisms regulating IgE production both for the development of successful schistosome vaccines and successful therapies for allergic diseases.

We have previously shown that schistosome infection intensity is negatively associated with allergen-specific IgE but is positively associated with schistosome-specific IgE responses [22]. Nonetheless, the relationship between sCD23 and schistosome infection intensity has not been widely studied. Therefore, the aim of the current study is to investigate whether sCD23 is associated with schistosome infection intensity and how levels of sCD23 relate to atopy (skin prick test and allergen-specific IgE) and levels of schistosome-specific IgE [22,23,24]. We focus on the monomeric 25-kDa sCD23 which is the most stable form of soluble CD23 [19] and hypothesise that the levels of sCD23 are negatively associated with atopy (measured as allergen-specific IgE titres or size of the whirl in a skin prick test) in schistosome-infected populations. To differentiate the effects of current schistosome infection from the effect of previous infection on sCD23 levels, we used a comparative study design whereby sCD23 levels are compared between two villages with different histories of schistosome infection. This study design has proved powerful in previous studies where the effects of host age on host immune responses [25] can be separated from the effects of cumulative history of infection in lifelong residents of endemic areas [22,23,24].

## Materials and Methods

### Study Design

The study was part of a larger programme investigating the immunoepidemiology, public health and control of schistosome infection in Zimbabwean villages. This study was comparative, comparing levels of sCD23 in people resident in a high schistosome infection area to those residing in a low schistosome infection area (schistosome levels designated according to World Health Organization guidelines [26]). Differences in infection levels reflect differences in infection transmission rates and thus different histories of infection amongst the villages [23]. Residents of the high infection village accumulate infection more rapidly, acquiring higher infection intensities at a younger age and consequently develop acquired immunity against the parasites earlier than their counterparts in the low infection village [23]. This study design was used to determine if these different histories of schistosome infection affected sCD23 levels.

### Study Population

A cohort of 434 participants was recruited from two villages, Magaya (high infection area (HIA), 17°63′ S, 31°91′ E) and Chitate (low infection area (LIA), 17°67′ S, 31°88′ E) villages in the Mashonaland East Province of Zimbabwe where *S. haematobium* is endemic. Magaya village is characterized by perennial rivers which lead to high transmission rates of schistosome parasites compared to Chitate village which is characterized by seasonal streams. In addition, households in Magaya are built along rivers, whereas in Chitate they are more dispersed and built further from the rivers as confirmed by GPS mapping of the area. Human contact with water harboring cercariae, the infective stage of schistosomes, is frequent (as assessed by questionnaire studies) in this area due to insufficient safe water and sanitation facilities. The demographic composition of this cohort is detailed in table 1. All participants in this study were lifelong residents of the villages and had provided at least 2 stool and 2 urine specimens for the diagnosis of helminth infections. They were all egg-negative for geo-helminths and *S. mansoni*, and had not previously received anti-helminthic treatment. No *Plasmodium* parasites were detected in the blood of the participants (this area is mesoendemic for malaria as detailed previously [27]). Thirty-three participants were HIV positive and the potential confounding effects of HIV infection [9,28] were accounted for in all statistical analyses.

### Ethical Statement

Permission to conduct the study was obtained from the Provincial Medical Director, while institutional and ethical approval was received from the University of Zimbabwe and the Medical Research Council of Zimbabwe, respectively. All participants had the aims and procedures of the project explained, and written consent was obtained before enrolment into the study. For young children, written assent was obtained from parents/guardians. At the end of the study, all participants were offered treatment with the recommended dose (40 mg/kg of body weight) of the anti-helminthic drug praziquantel.

### Immunological Assays

A maximum of 10 ml venous blood was collected from each participant into silicon-coated tubes without anticoagulant and serum was extracted for enzyme-linked immunosorbent assays (ELISA) to quantify antibody levels.

To determine the levels of sCD23, microtitre plates were coated overnight with 100 µl/well of capture antibody (anti-CD23, R&D Systems, cat. No. MAB1231) at 1 µg/ml in carbonate-bicarbonate buffer. Plates were washed and blocked with 2% BSA/PBS/3%/tween20 for 2 h before the addition of samples. Serum samples were diluted at 1:2 in 2% BSA/Tween 20 together with the standard (R&D Systems, cat. No. 123-FE). The secondary antibody, biotinylated anti-human CD23, aa 150-321, R&D Systems, cat. No. BAF123, which detected the 25-kDA monomer of sCD23, was added at 0.2 µg/ml. 100 µl/well of streptavidin-horseradish peroxide (GE Healthcare) at 1:6,000 were added after 4 washes and plates were incubated for 2 h at 37°C. For the total IgE, plates were coated overnight with 5 µg/ml of the capture antibody (anti-IgE, Dako), serum samples were diluted at 1:50, and the secondary antibody (anti-human horseradish peroxide-conjugated IgE, Sigma) diluted at 1:1,000. For allergen-specific IgE, plates were coated with 50 µl/well of affinity purified *Dermatophagoides pteronyssinus* (house dust mite, Der p 1) allergen (Indoor Biotechnologies) at 5 µg/ml in carbonate-bicarbonate buffer. Serum samples were diluted at 1:10 and secondary antibody (anti-human horseradish peroxide-conjugated IgE, Sigma) at 1:1,000. For anti-schistosome IgE responses, plates were coated with 5 µg/ml for cercarial antigen (CAP) and soluble worm antigen (SWAP) and 10 µg/ml for soluble egg antigen (SEA); serum samples were diluted at 1:20 and secondary antibody (anti-human horseradish peroxide-conjugated IgE, Sigma) at 1:1,000 for SEA and CAP, and 1:250 for SWAP.

For all assays, the substrate (ABTS, Southern Biotech) was used for the colorimetric reaction and the optical density read at 405 nm. The antibody concentrations were extrapolated from a series of titrations of standards (purchased from R&D Systems and NIBSC, respectively, for CD23 and IgE) which were run on each plate. The top standard concentration was 0.1 µg/ml for CD23 and 1,000 IU/ml for total IgE. Antibody levels were expressed as ng/ml and IU/ml, respectively, for CD23 and total IgE. Levels of Der p 1 – and schistosome-specific IgE responses were expressed as optical densities.

### Skin Prick Tests

As previously described [22], skin prick tests (SPT) were performed using a preparation of the house dust mite (HDM) *Dermatophagoides pteronyssinus*, grass mix, *Alternaria tenius,* and *Aspergillus fumigatus* (all from Diagenics), and positive responders (skin prick result equal or higher than the positive control) classified as atopic individuals. This aspect of the study involved a subgroup of 225 people (out of the 434) skin pricked (mean age: 21 years, range: 6-86; mean infection intensity: 0.7 eggs/10 ml urine, range: 0-28). The majority of atopic individuals were sensitised to the HDM, consistent with previous reports [29]. Therefore, the study focused on this allergen for both immunological assay and SPT to define atopy.

### Statistical Analyses

The study tested the effects of host factors on sCD23 levels as well as the relationship between sCD23 levels and skin prick reactivity. The association between sCD23 levels and levels of other serological IgE (total IgE, allergen (Der p 1)-specific IgE and schistosome-specific IgE) was also determined.

Initially, infection intensity and prevalence were compared between the two villages using the general linear model (GLM) and χ^2^ tests, respectively. In the GLM, infection intensity was the dependent variable while sex, age, HIV status and village (HIA and LIA), were the independent categorical variables. Age was categorised into three age-groups (6-10, 11-15 and ≥16 years) reflecting epidemiological patterns of infection, i.e. ages 6-10, infection is rising; ages 11-15, infection is peaking and ages 16 and above, infection is declining. Infection intensity was log_10_(x + 1) transformed to fulfil the assumptions of parametric tests conducted [30]. All the statistical analyses were performed in PASW statistics 17 (formerly SPSS) and test statistics were considered significant at p < 0.05.

To determine the host attributes related to levels of sCD23, an ANOVA was used with CD23 levels (square root transformed) as dependent variable and the host factors, host residential area (categorical, HIA/LIA), sex (categorical, male/female), age (categorical, 6-10/11-15/≥16), HIV status (categorical, HIV negative/HIV positive) and infection status (categorical, infected/uninfected) or infection intensity (continuous, log_10_(x + 1) transformed) as independent variables. To calculate the F ratio in the ANOVA, sequential sums of squares were used and the independent variables were entered in the order they are listed above to allow for the potential confounding effects of all the other variables before testing the effects of schistosome infection intensity [31]. β coefficients were used to determine the direction of the association.

To establish whether atopic and nonatopic individuals (determined by SPT) exhibited differences in their antibody levels (total IgE and sCD23), a binary logistic regression analysis was performed, using skin prick status as the dependent variable and sex, age, village, infection intensity, HIV status, and sCD23 or IgE levels (square root transformed) as predictors.

To determine whether sCD23 levels were related to levels of the serological levels of IgE (total IgE, Der p 1-specific IgE, schistosome-specific IgE), a MANOVA was conducted using each of the IgE variables (square root transformed) as the dependent variable and sCD23 (also square root transformed) as the independent variable after allowing for the potential effects of host residential area, sex, age, HIV status, and infection intensity with all independent variables appropriately transformed or categorised as before.

## Results

### Epidemiological Patterns of S. haematobium Infection

**Fig. 1:**
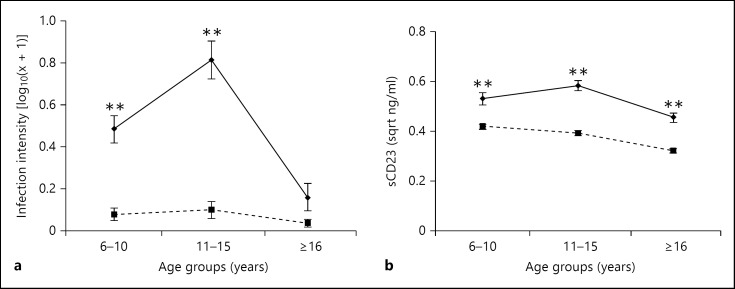
Age profiles of the study villages. Geometric mean infection intensity (**a**) and sCD23 (**b**) are plotted against age groups in the HIA (plain line) and the LIA (dotted line). Bars represent standard error of the means and asterisks represent significant differences in each age group between the two villages at ** p < 0.01.

Infection intensity and prevalence were significantly higher in Magaya, the HIA, than in Chitate, the LIA (23.2 vs. 0.6 eggs/10 ml; F_1, 428_ = 63.295; p < 0.001 and 49.8 vs. 8.06%; χ^2^ = 90.743; p < 0.001, respectively). There was a significant age and village interaction (F_2, 426_ = 10.466; p < 0.001). As shown in figure 1a, infection intensity rose to a higher level and peaked at a younger age in the HIA compared to the LIA, a pattern known as a ‘peak shift’ [23,32].

### Schistosome Infection Intensity Affects Levels of sCD23

**Fig. 2:**
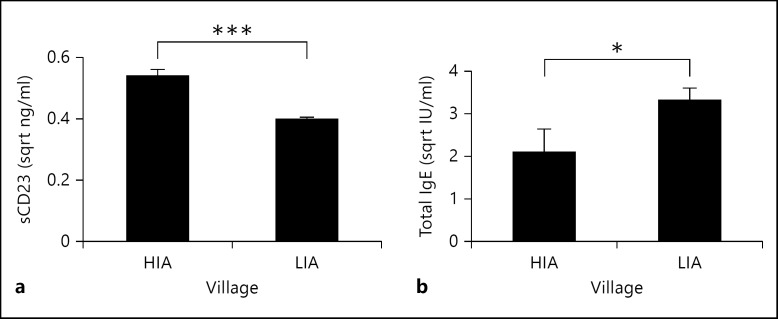
Comparison between the high infection and low infection areas. The mean of the square root-transformed concentrations of soluble CD23 (**a**) and total IgE (**b**) are plotted for the HIA and the LIA. Bars represent standard error of means and asterisks represent significant p values at * p < 0.05 and *** p < 0.001.

There was a significant association between place of residence and sCD23 levels as shown in table 2. Post-hoc analyses showed that overall, levels of sCD23 were significantly higher in the HIA compared to the LIA (t = 10.659, p < 0.001; fig. 2a). Comparison of total IgE levels between the two areas showed that levels of these antibodies were higher in the LIA (t = −2.08, p = 0.038; fig. 2b). After allowing for the confounding effect of residential area, sex, age group and HIV status, ANOVA showed a significant effect of schistosome infection intensity on sCD23 levels, with sCD23 levels increasing with schistosome infection intensity. Schistosome infection status did not show a significant relationship with sCD23 level (F_1, 427_ = 1.908, p = 0.168). Although sCD23 levels varied with host age, following the age-infection profile (fig. 1b), the interaction between age and residential village was not significant indicating that the relationship between sCD23 levels and age did not depend on the residential area.

### Atopy Is Negatively Associated with Levels of sCD23

**Fig. 3:**
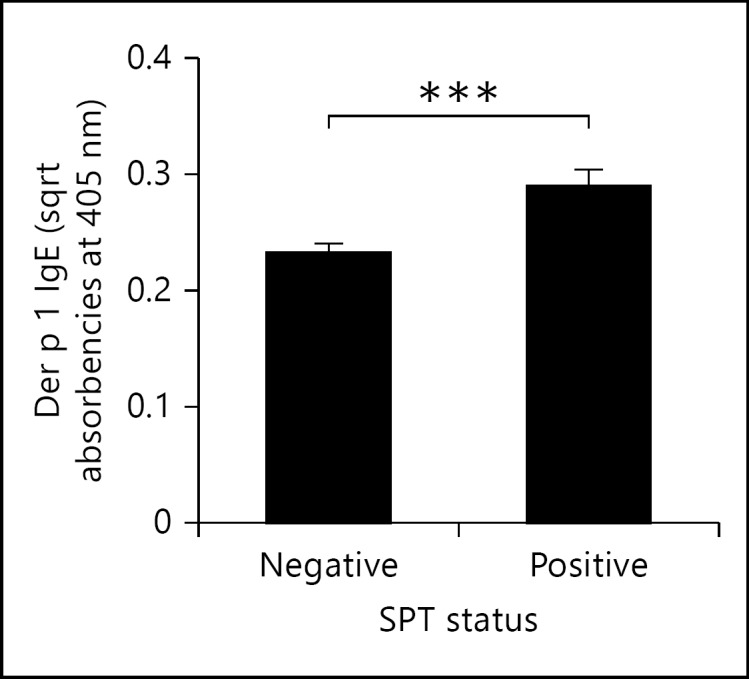
Comparison of anti-Der p 1 IgE antibodies in SPT-positive versus SPT-negative people. Mean levels of Der p 1 IgE (square root-transformed optical densities) are plotted for atopic and nonatopic groups. The p value (*** p < 0.001) was obtained from a contrast GLM allowing for the effects of host gender, age, HIV status, village of residence and infection intensity.

**Fig. 4:**
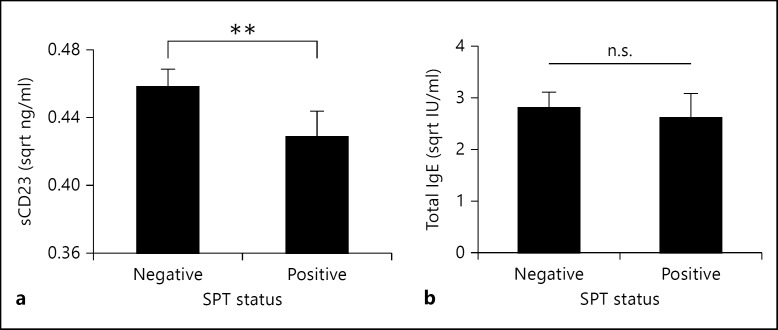
Comparison of sCD23 and total IgE levels in the study population. The mean of the square root-transformed concentrations of sCD23 (**a**) and total IgE (**b**) are plotted for SPT-positive and -negative individuals. Bars represent standard errors of the means. ** p < 0.01 obtained from GLM analysis allowing for the effects of host sex, age, HIV status, village and infection intensity. n.s. = Not significant (p > 0.05).

We initially established the relationship between SPT reactivity and levels of anti-Der p 1 IgE. This analysis showed that SPT-positive people had significantly higher levels of the antibodies (fig. 3). However, a binary logistic regression showed that atopic individuals (as determined by SPT), had significantly less sCD23 levels (B = −7.32, Wald = 7.663, p = 0.006; fig. 4a) but levels of total IgE did not differ between atopic and nonatopic individuals (B = 0.018, Wald = 0.032, p = 0.858; fig. 4b).

### Dissociation between Total IgE and CD23

**Fig. 5:**
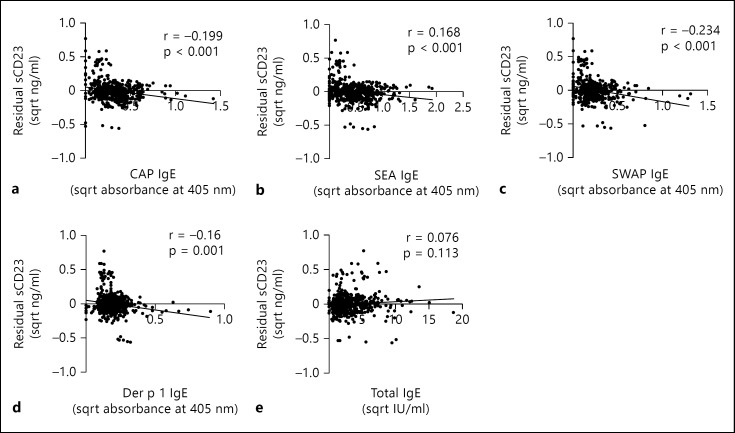
Relationship between sCD23 and IgE antibodies. Residuals (after allowing for age, sex, HIV status, village and infection intensity) of the square root-transformed concentrations of sCD23 are plotted against anti-schistosome IgE responses (square root-transformed optical densities) (**a-c**), anti-Der p 1 IgE (**d**) and total IgE (**e**). Significant results at p < 0.05 are highlighted in bold.

Analysis of the relationship between sCD23 levels and serological levels of IgE (total IgE, parasite-specific IgE and Der p 1-specific IgE) showed that parasite-specific IgE declined with increasing sCD23 levels as shown in table 3 and figure 5a–c. This association was significant for all parasite-specific IgE responses, i.e. anti-CAP IgE, anti-SEA IgE and anti-SWAP IgE. In addition, levels of sCD23 were significantly negatively associated with levels of anti-Der p 1 IgE (fig. 5d), but showed no significant association with total IgE levels as detailed in table 3 and figure 5e.

## Discussion

Our study investigated the relationship between serological levels of the sCD23 receptor and IgE levels in people exposed to schistosome infection. Overall, the study showed that sCD23 levels were negatively associated with antigen-specific IgE levels, and this was true for both schistosome-specific IgE levels and allergen-specific IgE. Interestingly, sCD23 levels showed no association with polyclonal IgE measured as total IgE. Of the different fragments of sCD23 released following cleavage of membrane-bound sCD23, our assay detected the most stable form, the 25-kDa fragment.

The data presented show that levels of sCD23 are significantly higher in the high *S. haematobium* infection area than the low infection area. Since the populations are similar in ethnicity and have comparable exposure background to other endemic infections such as malaria, with exposure to schistosome infection being the only differing characteristic, this result suggested that the difference in schistosome infection may contribute to differences in sCD23 levels. The lack of a peak shift in the levels of sCD23 suggests that the differences in levels of sCD23 in residents of the different villages are due to current rather than cumulative history of infection. Furthermore, analysis at the individual level demonstrated a significant positive association between levels of sCD23 and current schistosome infection intensity regardless of host village of residence. Interestingly, this association was lost if the level of the infection burden (infection intensity) was replaced by infection status, demonstrating that it was the burden of infection rather than infection status that drove the association with sCD23 levels.

Similar to findings by other groups showing a negative association between helminth infection and atopy [33,34,35,36], in this population schistosome infection intensity was negatively associated with atopy [22], so we related levels of sCD23 to skin prick reactivity. This revealed that SPT-positive individuals exhibit significantly lower levels of sCD23 than their SPT-negative counterparts. One potential mechanism for this relationship would be inhibition of antigen-specific IgE synthesis through the cleavage of the CD23 molecule into small soluble monomer fragments with downregulatory effects on IgE synthesis [16,20] or inhibition of the trimerisation of these monomer fragments [37].

This result contrasted with findings in asthmatic patients where levels of sCD23 were significantly elevated in asthmatic compared to healthy controls [38]. However, these authors also showed that sCD23 and monocytes expressing CD23 were elevated in patients with intrinsic asthma (i.e. nonallergic), but that these, in contrast to allergic patients, did not translate into elevated levels of IgE, nor positive SPT. In the current study, SPT-positive individuals had significantly higher levels of Der p 1-specific IgE than their SPT-negative counterparts, suggesting an association between the levels of sensitisation and the clinical manifestation of atopy in this population.

The role for CD23 in suppressing atopy has been investigated in mouse experimental models. For instance, CD23 has been shown to have an inhibitory role in the development of allergic airway inflammation and airway hyperresponsiveness [21]. Furthermore, CD23 has been implicated in the suppression of airway inflammation in mice chronically infected with the helminth parasite *Heligmosomoides polygyrus*[39]. A strain of mice naturally overexpressing IgE responses has also been shown to possess a variant CD23 present at low levels on B cells, which fail to trimerise and bind IgE [37]. How these functions of bound CD23 molecules are related to the soluble forms remains to be ascertained. In addition, unlike in humans, murine CD23 does not have the C-terminal tail that binds CD21 [15], so that these findings may not fully translate to the physiological function of sCD23 in humans.

In the current study, levels of polyclonal IgE were significantly higher in the LIA than the HIA, and were not significantly different between atopic and nonatopic groups. While there was no significant association between levels of sCD23 and total IgE, there were significant negative associations between sCD23 and the antigen-specific IgE levels. In a study of bronchial asthmatic patients, sCD23 levels were found to be significantly positively correlated to the total IgE levels in both the asthmatic patients and non-asthmatic controls [40]. One possible explanation for the differences in the two studies is that our study population was exposed to schistosome infection (which similar to previous reports [41] was positively associated with levels of polyclonal IgE).

Studies in *Leishmania chagasi* have shown that parasites can down-modulate CD23 expression on human B cells and macrophage cell lines, in part by increasing the release of sCD23 [42]. The release of sCD23 was dependent on the dose of the parasite, with increasing parasite doses resulting in increased levels of sCD23. This pattern is consistent with the positive association we observed between schistosome infection intensity and levels of sCD23. Mechanistic studies of CD23/sCD23 dynamics in human cells exposed to schistosome antigens will determine if a similar phenomenon occurs in the helminth parasites.

The negative association between the antigen-specific IgE levels and sCD23 is consistent with reports from other published work showing that monomeric sCD23 fragments decrease IgE secretion by PBMCs [18,19,20]. However, given that similar to other monomeric sCD23 fragments, the 25-kDa monomer of sCD23 has pleiotropic effects on several cell types [11,43], some of which are IgE-independent [44], the relationships observed here may not be directly causal but may be the result of several pathways and interactions between different cell types. Therefore, detailed mechanistic and pathway studies are required to determine if these associations are causative or confounding. Overall the study raises an interesting question as to why the sCD23 levels show a relationship with schistosome-specific IgE and with allergen-specific IgE and associated skin prick reactivity but not with polyclonal IgE. Understanding the regulation of antigen-specific IgE synthesis may contribute towards the development of safe helminth vaccines as well as immune therapies for atopic diseases.

## Disclosure Statement

The authors declare no financial or personal conflict of interest which may interfere with the study outcome.

## Figures and Tables

**Table 1:** Demography and infection levels of the study populations

		Age groups, years		
		6–10	11–15	≥16
Magaya:	Number	103	86	34
HIA	Mean age, years	8.2	12.5	38.8
	Sex ratio, M:F	50:53	37:49	14:20
	Mean infection^a^	17.2	39.1	2.4
	Prevalence, %^b^	51.4	61.4	23.5
	HIV-positive cases	4	4	6

Chitate:	Number	77	51	83
LIA	Mean age, years	8.5	12.3	39
	Sex ratio, M:F	38:39	24:27	11:72
	Mean infection^a^	0.76	1.03	0.22
	Prevalence, %	9	11.8	4.8
	HIV-positive cases	4	2	13

The sample sizes (n), mean age, sex ratio and schistosome infection levels are shown, for each village, for the 3 age groups used in all analyses.

a Geometric mean of schistosome infection (egg count per 10 ml urine);

b Prevalence of *S. haematobium* infection.

**Table 2:** Determination of factors affecting levels of sCD23 using ANOVA

Factor	d.f.	F value	p value
Village	422, 1	116	<0.001
Sex	422, 1	1.42	0.234
Age group	422, 2	13	<0.001
HIV status	422, 1	0.07	0.797
Schistosome infection intensity	422, 1	3.86	0.05^*^
Village and age interaction	422, 2	1.76	0.173
Village and infection intensity interaction	422, 1	0.519	0.471
Age and infection interaction	422, 2	0.017	0.983

* β coefficient = 0.028 (95% CI = 0.00012–0.057).

**Table 3:** Relationship between sCD23 and IgE levels

IgE	F value	p value	β coefficient (95% CI)
CAP IgE	18.74	**<0.001**	−0.233 (−0.339 to −0.127)
SEA IgE	12.94	**<0.001**	−0.368 (−0.568 to −0.167)
WWH IgE	27.98	**<0.001**	−0.250 (−0.344 to −0.157)
Der p 1 IgE	11.58	**0.001**	−0.093 (−0.147 to −0.039)
Total IgE	2.62	0.107	1.23 (−0.264 to 2.713)

MANOVA was conducted using each of the IgE variables (square root transformed) as the dependent variable and sCD23 (also square root transformed) as the independent variable after allowing for the potential effects of host residential area, sex, age, HIV status, and infection intensity. Significant p values are highlighted in bold. The degrees of freedom for all tests were 426, 1.

## References

[1] Black CL, Muok EM, Mwinzi PN, Carter JM, Karanja DM, Secor WE, Colley DG (2010). Increases in levels of schistosome-specific immunoglobulin E and CD23(+) B cells in a cohort of Kenyan children undergoing repeated treatment and reinfection with *Schistosoma mansoni*. J Infect Dis.

[2] Zhang Z, Wu H, Chen S, Hu L, Xie Z, Qiu Y, Su C, Cao JP, Wu Y, Zhang S, Wu G (1997). Association between IgE antibody against soluble egg antigen and resistance to reinfection with *Schistosoma japonicum*. Trans R Soc Trop Med Hyg.

[3] Hagan P, Blumenthal UJ, Dunn D, Simpson AJ, Wilkins HA (1991). Human IgE, IgG4 and resistance to reinfection with *Schistosoma haematobium*. Nature.

[4] Hagel I, Cabrera M, Buvat E, Gutierrez L, Santaella C, Borges R, Infante B, Salas MC, Barrios Y (2008). Antibody responses and resistance against *Ascaris lumbricoides* infection among Venezuelan rural children: the influence of ethnicity. J Trop Pediatr.

[5] Abbas AK, Lichtman AH (2009). Basic Immunology: Functions and Disorders of the Immune System.

[6] Bruynzeel-Koomen C, van der Donk EM, Bruynzeel PL, Capron M, de Gast GC, Mudde GC (1988). Associated expression of CD1 antigen and Fc receptor for IgE on epidermal Langerhans cells from patients with atopic dermatitis. Clin Exp Immunol.

[7] Bullock RJ, Barnett D, Howden ME (2005). Immunologic and clinical responses to parenteral immunotherapy in peanut anaphylaxis – a study using IgE and IgG4 immunoblot monitoring. Allergol Immunopathol (Madr).

[8] Busse WW, Rosenwasser LJ (2003). Mechanisms of asthma. J Allergy Clin Immunol.

[9] Mwinzi PN, Ganley-Leal L, Black CL, Secor WE, Karanja DM, Colley DG (2009). Circulating CD23+ B cell subset correlates with the development of resistance to *Schistosoma mansoni* reinfection in occupationally exposed adults who have undergone multiple treatments. J Infect Dis.

[10] Aberle N, Gagro A, Rabatic S, Reiner-Banovac Z, Dekaris D (1997). Expression of CD23 antigen and its ligands in children with intrinsic and extrinsic asthma. Allergy.

[11] Acharya M, Borland G, Edkins AL, Maclellan LM, Matheson J, Ozanne BW, Cushley W (2010). CD23/FcεRII: molecular multi-tasking. Clin Exp Immunol.

[12] Fournier S, Rubio M, Delespesse G, Sarfati M (1994). Role for low-affinity receptor for IgE (CD23) in normal and leukemic B-cell proliferation. Blood.

[13] Gordon J (1992). CD23 and B cell activation. Clin Exp Allergy.

[14] Gould HJ, Sutton BJ (2008). IgE in allergy and asthma today. Nat Rev Immunol.

[15] Conrad DH, Ford JW, Sturgill JL, Gibb DR (2007). CD23: an overlooked regulator of allergic disease. Curr Allergy Asthma Rep.

[16] Letellier M, Sarfati M, Delespesse G (1989). Mechanisms of formation of IgE-binding factors (soluble CD23) – I. Fc epsilon R II bearing B cells generate IgE-binding factors of different molecular weights. Mol Immunol.

[17] Lemieux GA, Blumenkron F, Yeung N, Zhou P, Williams J, Grammer AC, Petrovich R, Lipsky PE, Moss ML, Werb Z (2007). The low affinity IgE receptor (CD23) is cleaved by the metalloproteinase ADAM10. J Biol Chem.

[18] Bowles SL, Jaeger C, Ferrara C, Fingeroth J, Van De Venter M, Oosthuizen V (2011). Comparative binding of soluble fragments (derCD23, sCD23, and exCD23) of recombinant human CD23 to CD21 (SCR 1-2) and native IgE, and their effect on IgE regulation. Cell Immunol.

[19] Sarfati M, Bettler B, Letellier M, Fournier S, Rubio-Trujillo M, Hofstetter H, Delespesse G (1992). Native and recombinant soluble CD23 fragments with IgE suppressive activity. Immunology.

[20] McCloskey N, Hunt J, Beavil RL, Jutton MR, Grundy GJ, Girardi E, Fabiane SM, Fear DJ, Conrad DH, Sutton BJ, Gould HJ (2007). Soluble CD23 monomers inhibit and oligomers stimulate IgE synthesis in human B cells. J Biol Chem.

[21] Haczku A, Takeda K, Hamelmann E, Loader J, Joetham A, Redai I, Irvin CG, Lee JJ, Kikutani H, Conrad D, Gelfand EW (2000). CD23 exhibits negative regulatory effects on allergic sensitization and airway hyperresponsiveness. Am J Respir Crit Care Med.

[22] Rujeni N, Nausch N, Bourke CD, Midzi N, Mduluza T, Taylor DW, Mutapi F (2012). Atopy is inversely related to schistosome infection intensity: a comparative study in Zimbabwean villages with distinct levels of *Schistosoma haematobium* infection. Int Arch Allergy Immunol.

[23] Mutapi F, Ndhlovu PD, Hagan P, Woolhouse ME (1997). A comparison of humoral responses to *Schistosoma haematobium* in areas with low and high levels of infection. Parasite Immunol.

[24] Rujeni N, Nausch N, Midzi N, Mduluza T, Taylor DW, Mutapi F (2012). *Schistosoma haematobium* infection levels determine the effect of praziquantel treatment on anti-schistosome and anti-mite antibodies. Parasite Immunol.

[25] Fulford AJ, Webster M, Ouma JH, Kimani G, Dunne DW (1998). Puberty and age-related changes in susceptibility to schistosome infection. Parasitol Today.

[26] WHO Expert Committee (2002). Prevention and control of schistosomiasis and soil-transmitted helminthiasis. World Health Organ Tech Rep Ser.

[27] Imai N, Rujeni N, Nausch N, Bourke CD, Appleby LJ, Cowan G, Gwisai R, Midzi N, Cavanagh D, Mduluza T, Taylor D, Mutapi F (2011). Exposure, infection, systemic cytokine levels and antibody responses in young children concurrently exposed to schistosomiasis and malaria. Parasitology.

[28] Black CL, Mwinzi PN, Muok EM, Abudho B, Fitzsimmons CM, Dunne DW, Karanja DM, Secor WE, Colley DG (2010). Influence of exposure history on the immunology and development of resistance to human schistosomiasis mansoni. PLoS Negl Trop Dis.

[29] Sibanda EN (2003). Inhalant allergies in Zimbabwe: a common problem. Int Arch Allergy Immunol.

[30] Barnard C, Gilbert F, McGregor P (2007). Asking Questions in Biology.

[31] Mutapi F, Roddam A (2002). p values for pathogens: statistical inference from infectious-disease data. Lancet Infect Dis.

[32] Woolhouse ME (1998). Patterns in parasite epidemiology: the peak shift. Parasitol Today.

[33] van den Biggelaar AH, van Ree R, Rodrigues LC, Lell B, Deelder AM, Kremsner PG, Yazdanbakhsh M (2000). Decreased atopy in children infected with *Schistosoma haematobium*: a role for parasite-induced interleukin-10. Lancet.

[34] van den Biggelaar AH, Lopuhaa C, van Ree R, van der Zee JS, Jans J, Hoek A, Migombet B, Borrmann S, Luckner D, Kremsner PG, Yazdanbakhsh M (2001). The prevalence of parasite infestation and house dust mite sensitization in Gabonese schoolchildren. Int Arch Allergy Immunol.

[35] Flohr C, Tuyen LN, Quinnell RJ, Lewis S, Minh TT, Campbell J, Simmons C, Telford G, Brown A, Hien TT, Farrar J, Williams H, Pritchard DI, Britton J (2010). Reduced helminth burden increases allergen skin sensitization but not clinical allergy: a randomized, double-blind, placebo-controlled trial in Vietnam. Clin Exp Allergy.

[36] Yazdanbakhsh M, Kremsner PG, van Ree R (2002). Allergy, parasites, and the hygiene hypothesis. Science.

[37] Lewis G, Rapsomaniki E, Bouriez T, Crockford T, Ferry H, Rigby R, Vyse T, Lambe T, Cornall R (2004). Hyper IgE in New Zealand black mice due to a dominant-negative CD23 mutation. Immunogenetics.

[38] Sanchez-Guerrero I, Albaladejo MD, Garcia-Alonso AM, Muro M, Hernandez J, Alvarez MR (1994). Soluble CD23 (sCD23) serum levels and lymphocyte subpopulations in peripheral blood in rhinitis and extrinsic and intrinsic asthma. Allergy.

[39] Wilson MS, Taylor MD, O'Gorman MT, Balic A, Barr TA, Filbey K, Anderton SM, Maizels RM (2010). Helminth-induced CD19+CD23hi B cells modulate experimental allergic and autoimmune inflammation. Eur J Immunol.

[40] Lorenzo GD, Mansueto P, Melluso M, Morici G, Cigna D, Candore G, Caruso C (1996). Serum levels of total IgE and soluble CD23 in bronchial asthma. Mediators Inflamm.

[41] Hagel I, Lynch NR, Perez M, Di Prisco MC, Lopez R, Rojas E (1993). Modulation of the allergic reactivity of slum children by helminthic infection. Parasite Immunol.

[42] Noben NN, Wilson ME, Lynch RG (1994). Modulation of the low-affinity IgE Fc receptor (Fc epsilon RII/CD23) by *Leishmania chagasi*. Int Immunol.

[43] Gould HJ, Sutton BJ, Beavil AJ, Beavil RL, McCloskey N, Coker HA, Fear D, Smurthwaite L (2003). The biology of IgE and the basis of allergic disease. Annu Rev Immunol.

[44] Armant M, Ishihara H, Rubio M, Delespesse G, Sarfati M (1994). Regulation of cytokine production by soluble CD23: costimulation of interferon gamma secretion and triggering of tumor necrosis factor alpha release. J Exp Med.

